# Natural and Synthetic Agents Targeting Reactive Carbonyl Species against Metabolic Syndrome

**DOI:** 10.3390/molecules27051583

**Published:** 2022-02-27

**Authors:** Tapan Behl, Amit Gupta, Sridevi Chigurupati, Sukhbir Singh, Aayush Sehgal, Vishnu Nayak Badavath, Ahmad Alhowail, Vasudevan Mani, Saurabh Bhatia, Ahmed Al-Harrasi, Simona Bungau

**Affiliations:** 1 Chitkara College of Pharmacy, Chitkara University, Rajpura 140401, India; amitgupta2508@gmail.com (A.G.); sukhbir.singh@chitkara.edu.in (S.S.); aayushsehgal00@gmail.com (A.S.); vishnu.badavath@chitkara.edu.in (V.N.B.); 2 Department of Medicinal Chemistry and Pharmacognosy, College of Pharmacy, Qassim University, Buraidah 52571, Saudi Arabia; s.chigurupati@qu.edu.sa; 3 Department of Pharmacology and Toxicology, College of Pharmacy, Qassim University, Buraidah 51452, Saudi Arabia; aalhowail@qu.edu.sa (A.A.); v.samy@qu.edu.sa (V.M.); 4 Natural & Medical Sciences Research Centre, University of Nizwa, Birkat Al Mauz, Nizwa P.O. Box 33, Oman; sbsaurabhbhatia@gmail.com (S.B.); aharrasi@unizwa.edu.om (A.A.-H.); 5 School of Health Science, University of Petroleum and Energy Studies, Dehradun 248007, India; 6 Department of Pharmacy, Faculty of Medicine and Pharmacy, University of Oradea, 410073 Oradea, Romania; 7 Doctoral School of Biomedical Sciences, University of Oradea, 410087 Oradea, Romania

**Keywords:** reactive carbonyls species, advanced glycation end products, sequestering agents, advanced lipo-oxidation end products, metabolism, metabolic syndrome

## Abstract

Reactive carbonyl species (RCS) may originate from the oxidation of unsaturated fatty acids and sugar in conditions of pathology. They are known to have high reactivity towards DNA as well as nucleophilic sites of proteins, resulting in cellular dysfunction. It has been considered that various pathological conditions are associated with an increased level of RCS and their reaction products. Thus, regulating the levels of RCS may be associated with the mitigation of various metabolic and neurodegenerative disorders. In order to perform a comprehensive review, various literature databases, including MEDLINE, EMBASE, along with Google Scholar, were utilized to obtain relevant articles. The voluminous review concluded that various synthetic and natural agents are available or in pipeline research that hold tremendous potential to be used as a drug of choice in the therapeutic management of metabolic syndrome, including obesity, dyslipidemia, diabetes, and diabetes-associated complications of atherosclerosis, neuropathy, and nephropathy. From the available data, it may be emphasized that various synthetic agents, such as carnosine and simvastatin, and natural agents, such as polyphenols and terpenoids, can become a drug of choice in the therapeutic management for combating metabolic syndromes that involve RCS in their pathophysiology. Since the RCS are known to regulate the biological processes, future research warrants detailed investigations to decipher the precise mechanism.

## 1. Introduction

**Figure 1:**
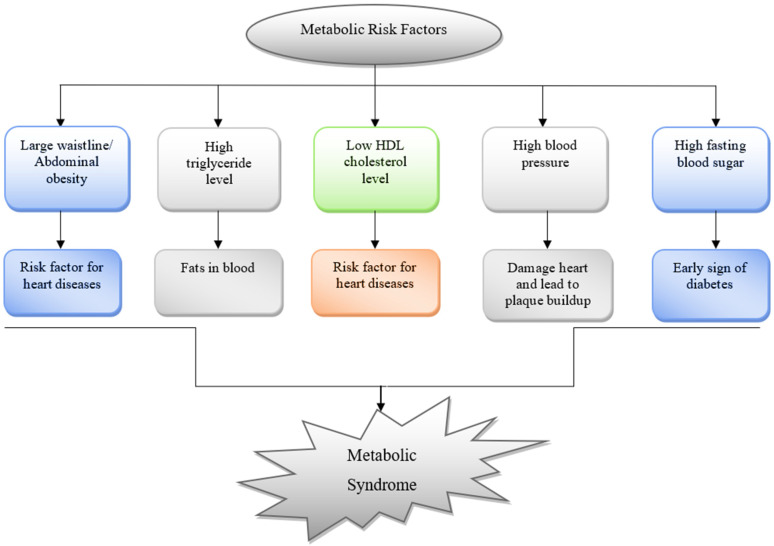
A Pictorial representation of Metabolic Risk factors along with their primary and secondary manifestations. These risk factors may serve as an early detection tool with secondary complications leading to the development of MeS. (HDL-High density Lipoprotein).

A metabolic syndrome (MeS) is a group of symptoms that occur simultaneously and is involved in the pathogenesis of cardiovascular complications including stroke and the development of type II diabetes as well. It is categorized as a collection of obesity-associated disorders that significantly enhance the risk of cardiovascular complications [1]. The biochemical properties involved in the body’s regular functioning are referred to as “metabolic”. Traits, conditions, or behaviors that enhance the risk of acquiring diseases are known as “risk factors” [2]. Various pathological conditions that may be described as metabolic risk factors are depicted in Figure 1. If a person is diagnosed with any of these risk factors, then that person will be considered to be at high risk for suffering from MeS [3]. Various risk factors constitute the diagnosis of MeS, which include high blood pressure, decreased levels of HDL (High density lipoprotein) in the body, increased levels of triglycerides, increased blood glucose level, and increased body surface area.

The combination of any of these risk factors mentioned in Figure 1 may lead to the diagnosis of MeS [3]. In case of the development of serious cardiovascular disorders, a person ought to have high blood pressure along with the additional factors mentioned in Figure 1, e.g., high blood fasting glucose levels or increased body surface area which may accelerate the development of these disorders [4]. So, in a nutshell, the presence of three or more factors is required to trigger any complications which may result in the development of various pathological conditions. From the review of studies and literature evidence, it can be concluded that MeS is associated with a higher risk of developing various disorders [5,6,7]. These may include cardiovascular complications, diabetes, obesity, osteoporosis, dyslipidemia, and disorders leading to the development of fatty acid build-up leading to atherosclerosis. Additionally, it has been studied that the underlying causes of the development of MeS are associated with obesity, insulin resistance, decreased physical activity, genetic factors, and advancing age [8].

With the increasing awareness regarding the risk associated with MeS, one can significantly reduce this risk by increasing physical activity and assuming a healthy diet rich in all nutrients that can manage to proactively regulate blood glucose, cholesterol levels, and blood pressure, leading to a decrease in complications associated with MeS [9]. In this review article, we have highlighted a few concerns which are associated with the development of MeS and can subsequently trigger various pathological conditions associated with the heart, vascular system, metabolic system, endocrine system, and even nervous system as well. The diagrammatic representation of these factors is depicted in Figure 1. With an increase in the number of metabolic risk factors, the risk for cardiac disease, diabetes, and even stroke is heightened. Being overweight and obese, along with a lack of physical exercise also increases the risk of developing MeS [4].

The majority of the literature available has focused on associating the activity of reactive oxygen species (ROS) and reactive carbonyl species (RCS) with specific complications like salt-related hypertension, obesity, and cardiovascular complications [10,11,12]. Moreover, there is literature available that focused on the role of ROS and RCS in the pathogenesis of MeS [13,14,15]. However, due to limited availability of data on preventive and treatment modalities for the prevalence of this mechanism of action, this review provides insights on the mechanistic approach for ROS and RCS in MeS.

In recent decades, an array of studies has been conducted to explore the role of using RCS sequestering agents, both natural and synthetic in nature, in order to combat the oxidative stress brought about in MeS and its related disorders like diabetes, obesity, dyslipidemia and osteoporosis. The scavengers used against them, and their general etiology has been elaborated further. However, there is not enough redressal on the areas of their applications. As MeS is a broad term with disorders sharing similar manifesting physiologies, it is possible that even without sufficient clinical data catering specifically to MeS patients or studies, agents that target a type of mechanism aimed towards another disorder can be used against MeS. By compiling the strategies of ROS and RCS implications in MeS, and studies that address individual disorders, this review aims to highlight the potential for the use of such agents across various MeS associated disorders. The role of RCS in most of the complications associated with MeS makes RCS a very viable target for disease management and therapy. However, certain knowledge gaps still persist, namely the exact mechanisms of actions of these agents that target RCS.

**Figure 2:**
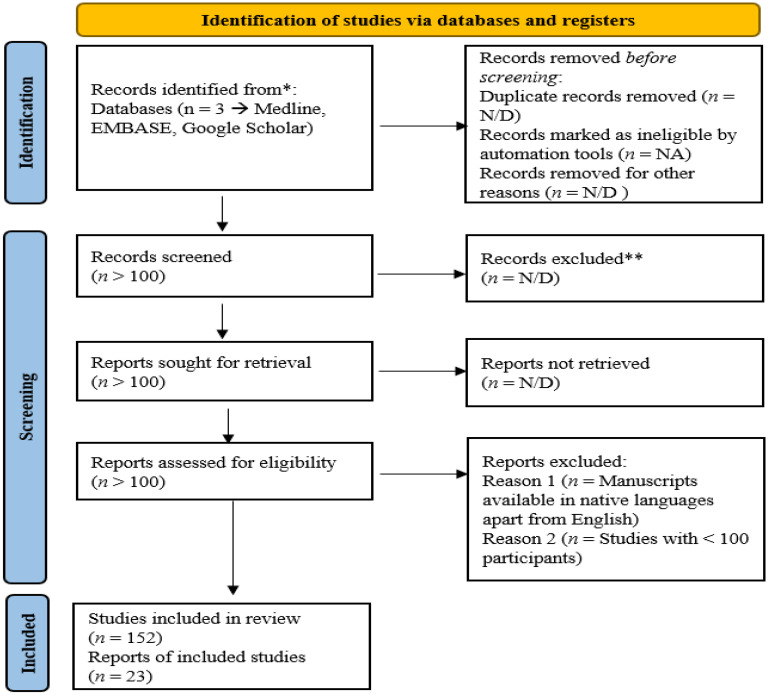
Counts of articles retrieved using PRISMA methodology for retrieval of articles. These included the numbers of articles retrieved through the process of identification, screening and thereby inclusion for the current study. *—all records included, research articles, review articles and meta-analysis reports, **—Conference abstracts were excluded.

This systematic review was carried out by running a search across the PubMed, MEDLINE and EMBASE databases along with Google Scholar for the terms “metabolic syndrome”, “reactive carbonyl species”, and study-specific manuscripts were obtained using their specific keywords. The screening was performed by assessing the relevance of the manuscript with regard to the motivation for this review and the PRISMA (Preferred Reporting Items for Systematic Reviews and Meta-Analyses) methodology, as mentioned in Figure 2 [16].

## 2. General Overview of Reactive Carbonyl Species (RCS)

MeS in general is characterized by an overproduction of ROS [17]. These ROS are necessary for homeostasis [18], however an imbalance in their concentrations leads to the accumulation of excess ROS, which are related to damaging changes across various molecules due to their reactive nature. They generate oxidative stress, which in turn leads to the progression of various pathophysiology of metabolic disorders [19]. Specifically, high levels of RCS are incredibly cytotoxic and induce cellular dysfunction. This leads to the progression and even acceleration of certain physiologies involved in MeS [20,21].

When ROS oxidizes the membrane lipids, they produce lipid peroxides (LOOH), which are then converted into different carbonyls such as aldehydes and ketones [22]. Among these, those having a carbonyl conjugated C-C bond with compelling biological functions are referred to as RCS, i.e., α-β unsaturated carbonyls) RCS are that class of biological molecules which are having one or more carbonyl groups that are frequently synthesized in numerous organisms and are primarily known for their damaging and toxic effects [23,24]. RCS can enter into body cells through industrial pollutants, external sources (medicinal chemical products, food additives etc.), and can also be produced endogenously (by the process of LOOH) [25].

Similar to other metabolic intermediates and their by-products, RCS are also known to exhibit electrophilic properties and are highly susceptible to several cellular constituents, the majority of which are nucleophilic [26,27]. It has been observed that unsaturated RCS are typically more reactive than saturated RCS, due to which unsaturated aldehydes, di, and keto-aldehydes are responsible for the majority of biological changes induced by RCS [28]. The best example for this would be the role of methyl glyoxal, a keto-aldehyde which is perhaps an example of some of the most potent glycating agents, reacting with proteins to form advanced glycation products, which result in loss of function of the proteins [29]. They generate oxidative stress by resulting in the formation of ROS, such as superoxides, peroxynitrites, and hydrogen peroxides and cause the release of pro-inflammatory cytokines, which initiate many diseases, including diabetes, vascular diseases, and dementia [30].

The most tempting targets for electrophilic attacks are the biomolecules with strong nucleophilic groups, e.g., thiol, imidazole, and hydroxyl groups. Dicarbonyls exhibit the properties to react with nucleophilic groups of macromolecules, such as proteins, nucleic acid, and amino-phospholipids, and cause irreversible alterations, resulting in the formation of an array of adducts and cross-linkages, which are known as an advanced glycation end products (AGEs) and advanced Lipoxidation end products (ALEs) respectively [28,31,32]. AGEs are produced due to glycoxidation, while ALEs are produced due to lipid peroxidation reactions. While AGEs have been studied extensively, ALEs are discussed less often. The salient difference is in their interactions with tissues. AGEs form stronger links with extracellular matrix and not are easily degraded, an example is Nε-(carboxymethyl)lysine (CML). This adduct once formed, is irreversible. The formation of Nε-(carboxymethyl)lysine (CML) involves a series of reactions that start from Amadori rearrangement to cross-linking. Due to these reactions, reactive oxygen species are generated. Hence, AGE formation directly contributes to exacerbation of oxidative stress [33]. 

ALEs are comparatively short-lived, however, over time start to accumulate as aging takes place [34]. Most examples of ALEs are aldehydes, and hence responsible for the loss of elasticity in cell membranes due to cross-linking. The reactions leading up to cross-linking also generate many RCS and ROS, and are hence directly responsible for increasing oxidative stress [35]. Therefore, while both AGEs and ALEs might be structurally different, their mechanisms of action are quite similar, and both of them are complexes that affect homeostasis.

ALE and AGE are the complexes that are poorly decomposed and get accumulated with age. They are ubiquitous in nature (blood and tissues) and exert a detrimental effect on the body [36]. ALEs and AGEs are known to be a primary source of RCS and ROS [37,38]. RCS primarily react at high rates with arginine, cysteine, and lysine residues. Due to the ubiquitous nature of amino acids spread across the protein sites, RCS is known to play an important role in the pathogenesis of various metabolic disorders, including hyperglycemia (especially in glucose tolerance conditions), obesity, increased B.P. (blood pressure), inflammation, and renal disorders including kidney failure [39].

**Figure 3:**
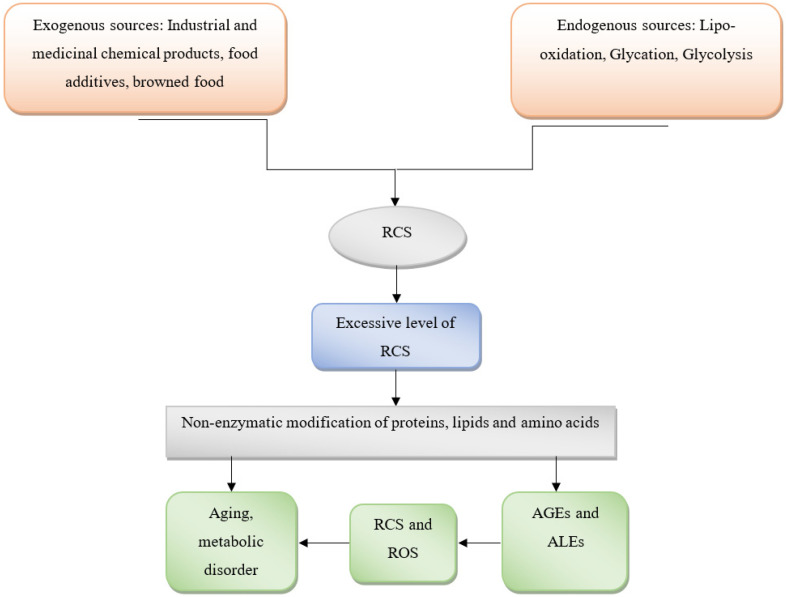
Mechanism of regulating metabolic dysfunction through RCS. RCS is traced to both exogenous and endogenous sources as listed here as a natural residence in the body. However, as soon as they are present in excess, they start catalyzing non-enzymatic modifications of proteins, lipids, and amino acids. The by-products of these reactions include AGEs, ALEs, RCS, and ROS, which further contribute to the pathogenesis of various disorders including MeS.

RCS can regulate homeostasis at many levels, most likely by destroying biological molecules and contributing to signaling/transcription control which is presented in Figure 3 [23]. Both the harmful and positive effects of reactive carbonyls are mediated through several signaling networks. Thus, RCS is known to have a dual activity which is mainly based upon its amount/quantity and time of action. RCS is known to exhibit an array of physiochemical and biological pathways through the process of cellular differentiation, cellular proliferation, and cell death (apoptosis and necrosis), cellular reproduction, metabolic homeostasis, immunological response, stress regulation (cellular), ageing [40]. Thus, a detailed understanding of the pathways mediated by RCS and its role in the pathogenesis of various disorders will provide an insight into its regulation and how the novel drugs have to be designed in order to effectively mitigate the pathological conditions associated with them [41].

## 3. The Role of RCS in Pathogenesis of MeS and MeS Related Disorders

**Figure 4:**
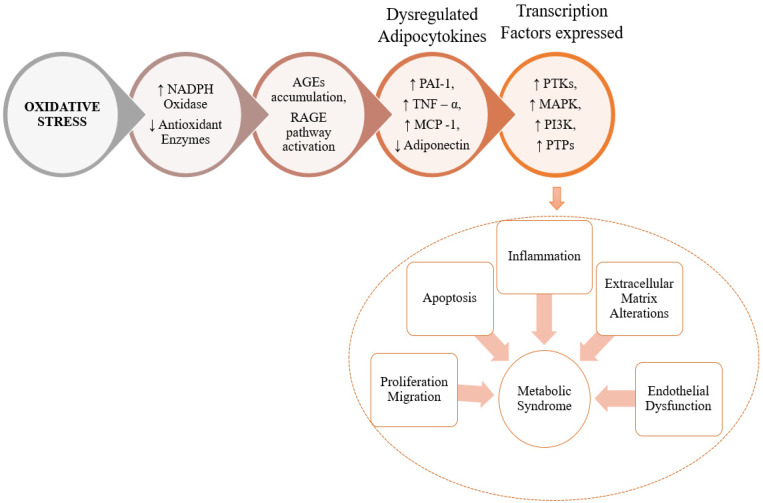
Pictorial representation of RCS exacerbate in oxidative stress leading to the development of MeS. The oxidative stress brought about by RCS increases the production of NADPH oxidase enzymes, which in turn start catalyzing the production of AGEs and RAGE (Receptor for AGE) pathway products. These products cause an accumulation of adipocytokines which catalyze the expression of transcription factors that cause inflammation, apoptosis, proliferation and other alterations. (NADPH = Nicotinamide Adenosine Dinucleotide Phosphate, AGE = Advanced Glycation End products, RAGE = Receptor for AGE, PAI-1 = Plasminogen Activator Inhibitor 1, TNF-Tumor Necrosis Factor, MCP = Methyl accepting Chemotaxis Proteins, PI3K = Phospho-Inositol Kinase, PTP = Protein Tyrosine Phosphatase, MAPK = Mitogen Activated Protein Kinase).

The term AGE is used when the ambushing RCS is obtained from sugar. However, in the case of ALEs, the attacking RCS is obtained from lipids. The structure and biological characteristics of AGEs and ALEs are similar and both are non-enzymatic in origin and covalently modified proteins [42]. Furthermore, some AGEs and ALEs have the same structure owing to the same precursors (such as carboxymethyl lysine, which is made up of glyoxal, generated by lipid and sugar oxidative degradation pathways) [43]. A detailed description of the role of RCS in oxidative stress resulting in cellular dysfunction is presented in Figure 4.

From the studies carried out in the past few decades, it was concluded that oxidative stress and protein carbonylation caused by RCS play a pivotal role in the pathogenesis of metabolic disorders, such as dyslipidemia, insulin resistance, as well as vascular and renal illnesses [44]. Hyperglycemia is associated with the disruption of the normal metabolic process, which is crucial for the production of reactive carbonyls, carbonyl stress, AGEs, and oxidative stress. Ryanodine receptors (RyR2) are involved in relaying Ca^2+^ signals in a variety of biological actions, including cardiac myocytes and airway smooth muscle. In diabetes, RCS changes RyR2 proteins, resulting in RyR2 dysfunction in both the heart and the lungs. Thus, the key to understanding the link between RCS and changed RyR2 in the cardiac origin and lungs in patients with diabetes could assist with the management of diabetes complications. Normal metabolic processes in patients with diabetes are disrupted by RCS leading to hyperglycemia [27]. Carbonyl/oxidative stress in diabetic complications is exacerbated by increased levels of RCS in the body. RyR are vital for modulating the smooth muscle of the airway. They play a pivotal role in the management of cardiac health, including contraction and relaxation. RyR become dysfunctional as a result of RCS, and RyR are engaged in critical pathways in diabetes complications [45,46]. 

The targeting of various RCS in cells, animal models, and humans to prevent metabolism syndrome is shown in Table 1 and is analyzed using various in-vitro and in-vivo animal models. These studies are carried out in the muscle cells, pancreatic cells, and even mesangial human cells to explore the potential of various compounds in the inhibition of AGEs, RCS, RAGE, and protein carbonyls to prevent metabolic diseases or syndrome [24]. 

Furthermore, various animal models were used to see how inhibiting the RCS, AGEs and RAEs affects metabolic diseases and their repercussions. The RCS sequestering activities of carnosine and its derivative have been studied using Zucker rats and ApoE (Apolipoprotein) deficient mice [47]. Furthermore, methylglyoxal (MG) injected Dahl salt-sensitive rats, STZ (Streptozotocin) induced diabetic rats, and CCl_4_-injected Wistar rats or Wistar rats fed with a high fructose-fed diet were used in diabetic complications by targeting RCS, AGEs, RAGE, and/or protein carbonyls [48]. In addition, T2D (Type-2 diabetes) patients were scrutinized in humans for elevated methylglyoxal levels and RAGE expression [7,45]. RCS and their adducts, on the other hand, are strongly linked to the advancement of metabolic illnesses and problems, which can be eased by RCS sequestering agents [7] (Table 1).

## 4. Management of Metabolic Disorders Using RCS

It has been considered that higher levels of RCS are responsible for the pathogenesis of various diseases and can even accelerate the ageing process [58]. However, lower quantities of reactive species can have positive effects. 

Endogenous enzymes: Various endogenous enzymes can metabolize (detoxify) highly hazardous RCS such as aldehydes produced by glycoxidation or lipid peroxidation. Various Phase I and Phase II reactions interplay and are involved in the metabolic machinery. These enzymes include CYP450 (Cytochrome P450), carbonyl reductase, glutathione-S-transferase, alcohol/ aldehyde dehydrogenases, and Aldo-keto reductase are responsible for the detoxification process [59]. 

Many plant-origin enzymes have been identified to possess an intrinsic mechanism to combat the generation of carbonyl species. In 2011, Yamauchi et al. identified the three prominent types of oxidoreductases that are present in plants, identifying their mechanism to be purely NADPH-dependent (Nicotinamide Adenine Dinucleotide Phosphate). The three types were: (i) aldehyde dehydrogenase which oxidizes aldehydes to carboxylic acid in the presence of NAD^+^, (ii) aldehyde reductase which reduces aldehydes or ketone groups to alcohols with NAD(P)H, and (iii) 2-alkenal reductase to reduce an RCS at its carbonyl conjugated C-C bond through NAD(P)H [60]. Similarly, the human body naturally possesses 17 types of ALDH enzymes that catalyze the metabolism of lipid-derived aldehydes. Townsend et al., identified the potential of one of these enzymes ALDH3A1, against HNE-induced apoptosis in the epithelial cells of cornea [61]. Studies have also exhibited that ALDH2-deficient individuals not only exhibit a higher risk towards cardiovascular diseases, but also Alzheimer’s disease [62]. This demonstrates the ability of ALDH2 to protect against chronic inflammation by metabolizing lipo-oxidation products. However, these enzymes have shown virtual to no protective activity against methylglyoxal, which is also one of the RAGE pathway products. 

CYP450 enzymes preferably metabolize RCS via hydroxylation or epoxidation. They have proven properties in oxidizing HNE, i.e., 4-Hydroxy 2-NonEnal, which has been implicated in tissue damage, aging, diabetes, multiple sclerosis, and other inflammatory associated conditions [63]. 

RCS scavengers: Over the past few decades various scavengers of reactive dicarbonyl species have been developed as an alternative therapy to suppress the adverse effects mediated by ROS without abolishing the normal physiological functions mediated by ROS. These scavengers act by direct binding to the functional groups of the primary nucleophiles which catalyze the rate-limiting step of ROS activity. Various in-vitro and in-vivo studies have been conducted and confirm that scavenging of RCS plays a pivotal role in the prevention of various pathological conditions. An example of this is the study by Y Guo et al. that demonstrated how diabetic retinopathy was reversed in rats after administration of carnosine. Moreover, glucose uptake in skeletal muscle cells was enhanced, which suggested reversal of glucolipotoxicity [64]. They also highlighted that this reversal was facilitated by the MAPK/ERK pathway, which is a pathway implicated in various pathogenetic mechanisms. Therefore, RCS scavengers may hold the key to preventing pathological conditions. Currently, 2-aminomethylphenols and carnosine analogs have been developed and shown promising results. However, further studies to explore novel molecules having better pharmacokinetic and pharmacodynamic properties might become next-generation compounds in combatting various MeS associated disorders [65]. 

One challenge with using scavengers is the requirement of adequate bioavailability, as they need to present in minimum a 1000-fold concentration to act on tissues. There is also a need for them to do so without toxicity.

Most of the scavengers are classified into different groups due to their ability to modify specific functional groups. For example, Thiol based scavengers like lipoic acid, and Amifostine can scavenge acrolein, while Imidazole-based scavengers target α,β-unsaturated carbonyl compounds. Similarly, 2-aminomethyl phenol can also be used as a dicarbonyl scavenger for methylglyoxal in plasma. Pyridoxamine or Pyridorin is also a hopeful drug of choice for the treatment of diseases with RCS-associated pathophysiology based on its ability to inhibit the formation of advanced glycation and lipoxidation products by scavenging free RCS [65].

Natural RCS scavengers: From the available literature, it can be suggested that eating a lot of fruits and vegetables lowers death from cardiovascular and other causes. In the case of chronic conditions, however, antioxidant supplements and multivitamins did not provide any benefits and, in fact, had the opposite impact. Natural products’ RCS-sequestering properties can be oppressed as a compelling strategy in the management of chronic diseases, based on the fact that they have the ability to sequester RCS like Carnosine [66].

L-Carnosine is perhaps the most prevalent naturally present RCS Scavenger in the brain and the muscles (~10 mM). Carnosine has the potential to scavenger intracellular RCS and result in the formation of unreactive covalent adducts. These adducts are thrown out of the body via urine, thus supporting RCS detoxification. Reports have proven that disorders like obesity and diseases, such as diabetes and Alzheimer’s, significantly reduce the amount of carnosine that is naturally present in the body, further increasing the susceptibility of such patients to accumulation of RCS [67]. Carnosines not only facilitate scavenging, but also mediate the physiologic pH, scavenge hydroxyl radicals, chelate redox metals, activate carbonic anhydrase, and stimulate nitric oxide synthesis. Carnosine possesses an unmatched ability to selectively quench α,β-unsaturated carbonyl compounds, reducing its toxicity [68].

Anserine (β-alanyl-L-methylhistidine) is another natural RCS scavenger that, like carnosine, detoxifies HNEs. It belongs to a family of Histidine dipeptides which have been proven useful in alleviating systemic oxidative and glycative stress. The bio-efficacy of these natural RCS scavengers is severely affected by a person’s genetic nature, and hence needs more studies on gene–nutrient interactions to conclusively establish a treatment strategy using them [65].

Natural products: In *ob*/*ob* (Obese) mice, black rice (which contains anthocyanin, Gamma-Aminobutyric acid (GABA), tocopherol, tocotrienols) was found to have anti-hyperinsulinemic and anti-hyperlipidemic effects. The effects/results of natural products were not limited to animal studies only, but they also exhibit effects in humans in the prevention of chronic illness. The initial step in identifying prospective candidates for target strategies to reduce OS-related chronic diseases is to test natural items for RCS quenchers. However, further research into gene–nutrient interactions is needed, as is improving the restricted bioavailability/bio-efficacy of natural compounds [66].

Anthocyanins have been to effectively prevent the formation of AGEs altogether by trapping methylglyoxal under simulated conditions. In addition, anthocyanin extracts have also been reported to have preventive effects on diabetes. They have been shown to lessen the symptoms of hyperglycemia and insulin sensitivity through AMP-activated protein kinase activation in rats. Their potential health benefits also include other oxidative stress combating activities, such as antioxidant, anti-inflammatory, antimicrobial, and anti-carcinogenic [69,70,71].

Emerging areas of nanotechnology have delved deep into improving the bioavailability of these natural compounds so as to overcome the natural hindrances posed by natural products. The use of nano-delivery systems can enhance their efficacy by significantly improving their bioavailability, as has been done in the case of Curcumin, one of the most notorious natural compounds in matters of bioavailability [65].

**Figure 5:**
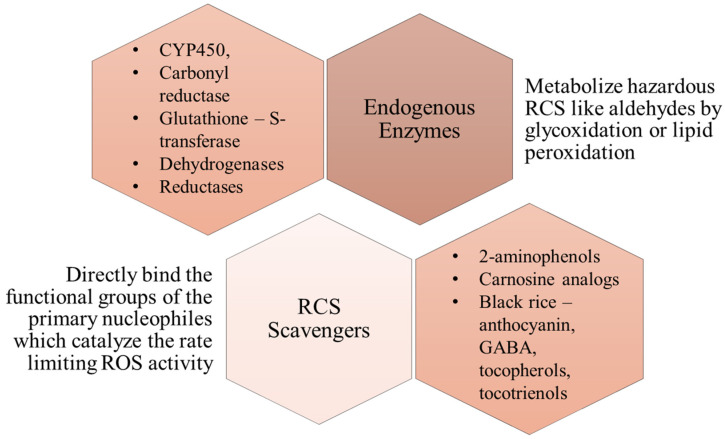
Reported strategies for combating the presence of RCS with their MoA (Mechanism of Action) and examples. These strategies lead the way to the development of potential therapeutic management approaches against MeS. These enzymes are both natural and synthetic in nature while RCS Scavengers are mostly natural in nature. Scavengers, due to their direct activity of binding with the interfering group itself, make a much more significant impact in managing RCS molecules. (CYP450 = Cytochrome 450, GABA = Gamma Amino Butyric Acid)**.**

Figure 5 depicts the mechanism of action in RCS scavenging and the role of endogenous enzymes in the development of potential therapeutic management against MeS (Table 2).

### 4.1. Synthetic Agents Targeting Carbonyl Species for Combatting MeS

**Figure 6:**
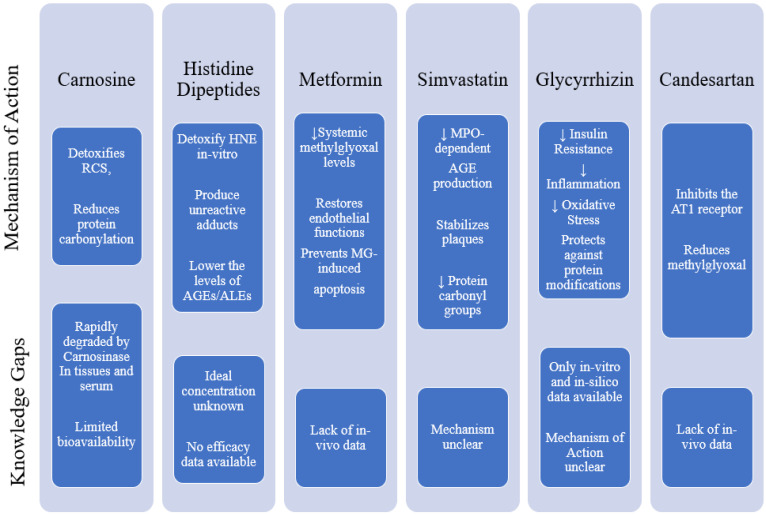
Synthetic agents targeting RCS for combating MeS. The figure depicts the mechanisms of action that have been identified for each therapeutic drug, supporting their roles in management of MeS, irrespective of their original indications. However, there still exist knowledge gaps which have also been identified and demonstrated. (RAGE = Receptor of AGE protein adducts, AGEs = Advanced Glycation End products, MG = Methylglyoxal, HNE = 4-Hydroxy-Trans-2-NonEnal, RCS = Reactive Carbon Species, ALEs = Advanced Lipid peroxidation End products, AT1 = Angiotensin 1, MPO = Myeloperoxidase)**.**

Various studies have been conducted to explore or develop numerous synthetic agents that have therapeutic effects by targeting carbonyl species for combating MeS. The agents are depicted in Figure 6 along with the research gaps from the available literature.

### 4.2. Carnosine

In both rodents and humans, carnosine detoxifies RCS, resulting in interesting positive benefits due to a reduction in protein carbonylation. Furthermore, despite the presence of hydrolytic enzymes (carnosinases), it is capable of preventing chronic carnosinemia. This activity has been identified in humans. L-carnosine, a naturally occurring dipeptide, can also scavenge intracellular RCS, generating unreactive covalent adducts [67,73] that are then eliminated in the urine [74], offering an alternative RCS detoxification route. Obesity has been linked to a decrease in naturally produced L-carnosine, which increases the risk of RCS buildup. In rat models, L-carnosine supplementation has been shown to reduce RCS. However, only a few human studies have been conducted [74,75,76,77,78]. Carnosine is used for various MeS, e.g., for obesity, dyslipidemia, type-II diabetes, and renal function [74] (Table 3).

Various studies are there, showing the use of carnosine in the management of different metabolism syndromes [72,79,80]. Because carnosine is rapidly degraded by the carnosinase enzyme in tissues and serum, its bioavailability is restricted, and carnosine-ACR adducts were found in all participants’ urine [74]. Low serum carnosine dipeptidase-1 (CNDP1) gene polymorphism protects patients with T2D against diabetic complications including nephropathy and can be allosterically blocked by the injection of reduced glutathione (GSH), N-acetylcysteine, and cysteine in diabetic mice [78].

### 4.3. Histidine Dipeptides

Histidine dipeptides like carnosine (-alanyl-l-histidine) and anserine (-alanyl-l-methyl histidine) have been found to successfully detoxify HNE in vitro by producing unreactive adducts [81]. The major reaction site of HNE adduction is histidine, which is considered the most receptive nucleophilic residue present in the proteins [82]. From the in-vitro studies in Zucker obese rats, it was concluded that decreasing the carnosine supplementation (histidine-dipeptides), as a result of the reduction of protein carbonylation and glycation, can significantly attenuate the pathogenesis of hypertension, renal damage, and dyslipidemia [83].

Histidine-dipeptides have also been shown to be effective in a variety of animal models with systemic oxidative and/or glycative stress. In these animal models, persuasive evidence can be drawn that histidine-dipeptides arbitrate the positive effects by lowering the levels of AGEs/ALEs, which results in the inhibiting AGEs/ALEs-RAGE damage [84,85,86,87].

Individuals with various genetic backgrounds may have varied bio-efficacy of supplements due to gene-nutrient interactions [88]. Vit. C-glutathione S-transferase and Vit. E-haptoglobin have both been linked to such interactions [89]. A link between low serum carnosine levels and diabetic nephropathy has also been discovered. Individuals with the specific alleles present on the CNDP1 (Carnosine DiPeptidase 1) gene had significantly higher serum carnosinase actions. Patients with diabetes having 5-5 alleles, who made up nearly a third of the population in this study, were shown to be less prone to kidney complications [90].

In a variety of illness scenarios where chronic oxidative or glycate stress is a prominent feature, histidine-dipeptides have been found to be helpful [84,91,92]. In these models, there is also persuasive evidence that histidine-dipeptides exert their health-promoting effects by lowering the levels of AGEs and ALEs, thereby inhibiting the detrimental AGEs/ALEs-RAGE axis. Furthermore, carnosine has been linked to probable involvement in blood glucose regulation via autonomic nerve control [93]. It is worth noting that type 2 diabetics have lower muscle carnosine levels [94]. The concentrations of carnosine in the blood and the quantity of cells in the pancreas were shown to have a significant relationship [95].

Although it was only demonstrated in an animal model, oral carnosine administration lowered plasma corticosterone levels and reversed stress-induced declines in glucose tolerance and glycogen content in the liver and muscle [96]. It is unknown what the ideal concentration of histidine-dipeptides is for eliciting positive effects. To date, no intervention studies have been conducted to assess the efficacy of histidine-dipeptide on MeS [97].

### 4.4. Metformin

Metformin is the first-line medication used for the treatment of type-II diabetes. It acts by reducing systemic methylglyoxal levels in type-II diabetes [98,99,100,101,102,103]. Methylglyoxal (MG) is a reactive alpha-dicarbonyl that is hypothesized to play a role in diabetes complications as a toxin or a precursor for AGEs. Methylglyoxal is an example of a keto-aldehyde that has been implicated in bringing about a vast majority of biological changes that are induced by RCS. Therefore, metformin holds great potential as an RCS scavenger in ameliorating MeS and MeS related disorders. Glyoxalase detoxifies it to D-lactate (DL) from triose phosphates. The effects of the diamino biguanide drug metformin and hyperglycemia on MG and its detoxifying products in type 2 diabetes (T2D) have been examined since guanidino compounds can block dicarbonyl groups [101]. 

Metformin lowers MG levels in a dose-dependent manner and lessens the impact of deteriorating glycemic control on MG levels. Metformin medication may protect against diabetes complications through mechanisms other than its antihyperglycemic impact, to the extent that increased MG levels contribute to their development [98,99].

Metformin has mostly been elucidated as an antidiabetic agent that improves insulin sensitivity but has shown effects in restoring endothelial function in rats. In the same study, it also significantly improved glycation and oxidative stress-related symptoms and enhanced nitric oxide bioavailability. Increased levels of pro-inflammatory CCL2 marker demonstrated its ability to improve on inflammatory symptoms as well [102]. Metformin has also been reported to prevent methylglyoxal-induced apoptosis in mouse Schwann cells, further implicating its role in management of diseases beyond diabetes [103].

### 4.5. Simvastatin

Simvastatin reduces MPO-dependent AGE production, which suppresses plaque RAGE expression. This impact may help to stabilize plaques by reducing the production of PGE2 (Prostaglandin E2)-dependent MMPs (matrix metalloproteinases), which are responsible for plaque rupture [104]. Simvastatin reduces myeloperoxidase-dependent AGE production, inhibiting macrophage RAGE expression. This effect, in turn, may help to stabilize plaques by blocking the release of PGE2-dependent MMPs that cause plaque rupture [105].

These roles focus on the use of simvastatin in treatment of Atherosclerosis and other cardiovascular diseases. It has been extensively studied to demonstrate antioxidant activity and enhance NO production in vasculature and reduction of inflammation, especially in the areas of bones, kidneys, and blood vessels [106]. With the increase in evidence of the role of oxidative stress in the etiology of diabetes, there have been studies that cross-link the use of antioxidant Simvastatin against diabetes [107]. They have demonstrated a significant drop in the level of protein carbonyl groups, a type of AGE product in T2DM patients treated with Simvastatin [105]. This suggests that simvastatin could protect against protein damage in such patients. Due to its proven effects against AGEs, Simvastatin can be chosen as a first-line treatment modality in preventing RCS escalations. Simvastatin was found to increase the T-SH groups in T2DM patients, implicating its role in protecting the functions of naturally present thiols and prevent damage done to them by free carbonyl species. The mechanism in question here is unclear. However, another study published by Aviram et al., does demonstrate that atorvastatin, another member of the statin family possesses antioxidant potential and protect fats from oxidation induced by free copper ions [108]. Detailed studies are required to elucidate whether the same mechanism is shared by simvastatin. 

### 4.6. Glycyrrhizin

In rats with MeS, glycyrrhizin, the main ingredient of licorice root, has been shown to have significant effects in patients with hyperglycemia by reducing insulin resistance and is even helpful in the management of dyslipidemia and obesity. This syndrome is linked to liver dysfunction. In fructose-fed rats, glycyrrhizin treatment reduced hepatic inflammation, oxidative stress and even cell death (apoptosis). The findings imply that glycyrrhizin could be used to treat hepatocellular damage in people with MeS [109].

Glycyrrhizin has also been implicated in protecting against diabetes, as suggested by a study conducted by Sahir Sultan et al. that demonstrated the role of GA in inhibiting the non-enzymatic glycation process in-vitro and in-silico. This study demonstrated the ability of glycyrrhizin to inhibit the formation of fructosamine and AGEs, thus inhibiting the AGE glycation process and reducing the oxidative stress in the cells. It also offered significant protection against protein modifications brought about by AGE glycation products by preventing secondary conformation formation, which may lead to accumulation of AGE products and exacerbate oxidative stress in the cells. This property of glycyrrhizin has significant potential as it demonstrates these properties regardless of the types of proteins involved. This makes glycyrrhizin a viable candidate for use in combating all disorders that have oxidative stress related implications. However, there is a lack of in-vivo data to support these assessments [110].

### 4.7. Candesartan

Methylglyoxal (MG) is a primary precursor of carbonyl stress and is found to be elevated in patients with chronic kidney disease (CKD). This precursor was also found to contribute to the advancement of hypertension, vascular injury, and kidney injury especially in patients with diabetic nephropathy [56]. Through an RCS-mediated mechanism, this chemical causes salt-sensitive hypertension. In Dahl salt-sensitive (Dahl S) rats, a rat model of CKD, the function of MG in the pathophysiology of hypertension and cardio-renal damage are investigated [111]. The findings suggest that MG is associated with hypertension, cardiac and renal damage, increased levels of inflammation, and elevated oxidative stress, all of which might be mitigated by using an ARB [112,113].

Candesartan is intrinsically an Angiotensin II receptor blocker, hence is capable of independently inhibiting the AT_1_ receptor. An increased amount of AT2 in the cells gives way to RAAS and LOX activation, along with an increase in oxidative species. These contribute to the oxidative stress and increase inflammation. The RAAS (renin–angiotensin–aldosterone system) pathway also triggers the neurohumoral activation, which in turn contributes to the development of metabolic syndrome. Thus, using an AT2 (angiotensin II) blocker like candesartan can effectively mitigate the inflammation in the cells, as well as prevent the endothelial dysfunction brought about by neurohumoral activation and remodeling of the vasculature [114].

## 5. Natural Agents Targeting Carbonyl Species for Combating MeS

Various disorders including diabetes, dyslipidemia, osteoporosis, obesity and MeS are the most common metabolic illnesses, which emerge when normal metabolic processes are disrupted [103]. Oxidative stress, Nrf2 (Nuclear Factor Erthryoid 2 Related Factor 2) pathways, epigenetics, and changes in mRNA (messenger RNA) expression constitute the most common pathophysiology of the following illnesses [115,116]. It is well known that a larger intake of fruits and vegetables reduces all-cause and cardiovascular mortality, although multivitamins and antioxidant supplements have been shown to have no benefit, if not even harm, in the prevention of chronic diseases [117,118,119]. The first step in identifying prospective candidates for a targeted blueprint to avoid oxidative stress-related chronic diseases is to screen natural items for RCS quenchers [120].

### 5.1. Natural Products Combatting MeS

Natural antioxidative compounds and Nrf2 activators have been shown to help people with MeS. An accumulating number of studies indicate that some natural products or molecules are able to modulate MeS and its risk factors and are presented in Figure 1. They hold a tremendous potential of natural and herbal compounds in the prevention and management of MeS and may counteract the increased morbidity associated with these syndromes. A wide array of literature is available that confirms these natural and herbal molecules can revamp MeS and its associated risk factors [121,122,123]. However, this wide array of literature is not very specific to either their mechanism of action or their activity in RCS scavenging or MeS amelioration. Therefore, only treatment molecules with enough evidence to support RCS scavenging activities have been included in this review, and they are grouped by the disease they have shown most activity against.

### 5.2. Diabetes

Chronic hyperglycemia is a complication of diabetes mellitus, which is primarily caused by a deficiency in insulin production and/or insulin action induced by the failure of islet β-cells of Langerhans and insulin resistance. Increased hepatic glucose synthesis and decreased peripheral glucose utilization are two other main abnormalities that contribute to the development of diabetes.

#### 5.2.1. Polyphenols

Aromatic natural compounds are the major characteristic of plant phenolics. There are various phytoconstituents, including anthocyanins and flavanols (flavones and isoflavones family), that contribute to this category. Plants rich in these phytoconstituents exhibits natural antioxidants activity. The mechanism involved in the activity is regulated by scavenging free radicals, activating antioxidant enzymes, chelation of heavy metals, boosting cell resilience and reciprocating the effects mediated by RNS (Reactive Nitrogen Species) and ROS. Various herbal plants such as *Hibiscus sabdariffa* (phenolic extract) are known to demonstrate modulation of mRNA activity by activity modulated through phytoconstituents, e.g., Ellagitannin, Quercetin, and epigallocatechin-3-gallate [124].

Flavonoids are a type of polyphenol that has been examined extensively for the prevention as well as treatment of MeS related risk factors [37]. They improve obesity related MeS by increasing the energy outflow and oxidation of fats. They also exert secondary effects by decreasing appetite and by absorption of glucose [125]. Flavonoids are also known to play an important role in decreasing hepatic inflammation, hepatic oxidative stress, and can boost insulin-mediated activities in the hepatic cells, skeletal cells, as well as in adipose tissue, all of which can help with insulin resistance [125,126].

#### 5.2.2. Nrf-2 Activators

To address oxidative stress-related illnesses, several activators of Nrf2 have been discovered. Various direct and indirect Nrf-2 activators have been identified which have shown promising results in the management of various metabolic disorders and might become a drug of choice as an adjuvant with the standard care of treatment. For. e.g., synthetic triterpenoid bardoxolone methyl and natural isothiocyanate sulforaphane, which is derived from broccoli sprouts, are two examples of such activators that are now being used in clinical trials [127,128]. In the case of diabetic patients, they were treated with glucose control, blood pressure control, lipid-lowering, and renin–angiotensin system blockade, but the development and progression of nephropathy and cardiomyopathy in diabetic patients remained unavoidable. Finally, these Nrf2 cross talks with other signaling pathways are of clinical importance in the etiology of many human disease conditions, especially those with very complicated multifactorial molecular interactions, such as diabetes complications [127].

The extra formation of ROS generated by hyperglycemia is thought to be the primary cause of these diabetes problems. The pathogenesis of diabetic nephropathy is aided by oxidative stress. NrF2 regulates cellular defense systems against oxidative stress by activating antioxidant gene transcription [128].

#### 5.2.3. Alpha-Lipoic Acid (ALA)

Octanoic acid is the source of α-lipoic acid, which is a dithiol molecule. Its antioxidative properties include free radical scavenging, metal ion chelation, and antioxidant recycling. This has been shown to exhibit pharmacological effects in the improvement of diabetic neuropathy, diabetic retinopathy, regulation of neural blood flow, and nerve conduction, which are confirmed by various in-vitro experimental animal models [129].

As an oral or intravenous medication, ALA should be regarded as a drug rather than a nutrient. There is strong evidence that ALA is beneficial for practically all ageing disorders, including heart disease and dementia. The evidence for ALA’s usefulness as a supplement to a healthy diet and lifestyle in prediabetics and diabetics (type 1 and 2) is impressive. The use of ALA intravenously for peripheral neuropathy has adequate proof to be approved in Germany. Those suffering from diabetic peripheral neuropathy have also found relief by oral supplements [130,131].

#### 5.2.4. Melatonin

Melatonin (5-methoxy-n-acetyl-tryptamine), a pineal endogenous hormone, can influence the activity of the immune system, restrict carcinogenesis, and decrease oxidative stress. It is a free radical scavenger that protects DNA, proteins, and membranes from free radical damage. Increased CAT (Catalase) activity, decreased hepatic GSH GSH (Gluthathione)-peroxidase activity, and decreased lipid peroxidation are some of the other outcomes [44]. Melatonin’s effects on insulin secretion are mediated by melatonin receptors, according to research (MT1 and MT2). Melatonin lowers insulin secretion by blocking the cAMP or cGMP pathways. Melatonin, on the other hand, has been demonstrated to stimulate the PLC/IP3 pathway, which mobilizes Ca^2+^ from intracellular reserves and enhances insulin release as a result.

Meanwhile, both in vivo and in vitro, insulin secretion has a circadian rhythm that appears to be created inside the islets and is impacted by melatonin by generating a phase shift in insulin secretion. The idea of limiting the effect of melatonin in islets is appealing, while an islet-specific attenuation of melatonin action may be necessary because systemic effects of an MT2 blockage are possible [132].

### 5.3. Obesity

Obesity is characterized as adipose tissue accumulation that is excessive and pathological. It is one of the world’s most serious public health issues, with an increasing prevalence that affects people of all ages and genders. Body mass index (BMI), which ranges from 25.0 to 30.0 kg/m^2^, is used to define overweight and obesity. Various mechanisms or factors are involved in the development of obesity which includes hereditary, environmental, and behavioral factors. Obesity is also associated with the development of various chronic disorders including diabetes, MeS, cardiovascular complications, malignancies, respiratory disorders, including COPD, asthma, and even obstructive sleep apnea.

#### 5.3.1. Terpenoids

Terpenoids are one of the most diverse groups of natural compounds. Lycopene is a member of the carotenoid subgroup of terpenoids. Lycopene is a potent antioxidant that is able to prevent LDL-C (low density lipoprotein–cholesterol) and LOOH. Hormonal and immune system regulation, anti-angiogenesis, cell proliferation inhibition, apoptosis induction, reduction of pro-inflammatory marker generation, and reduction of tumor necrosis factor (TNF)-mediated induction of pro-inflammatory indicators are among its other biological effects [133].

Plants have a tremendous potential to synthesis a wide range of terpenoids, especially when the terpenoid biosynthetic route is combined with other secondary metabolic pathways. Tocopherol biosynthesis, for example, is the consequence of a combination of the shikimate and isoprenoid pathways, which lead to homogentisic acid phytyl diphosphate, which in turn leads to the development of tocopherols [134].

#### 5.3.2. Organosulfur

Bioactive chemicals, such as allicin, allixin, and allylsulfides, are prevalent in organosulfur compounds found in Allium plants such as garlic and onion. Garlic and onions have anti-obesity effects, such as decreasing cholesterol synthesis via hepatocytes by inhibiting HMG-CoA (β-Hydroxy β-methylglutaryl-CoA) reductase, preventing the platelet aggregation, inhibiting inflammatory enzyme activity, decreasing iNOS (Inducible Nitric Oxide Synthase) expression in macrophages, and decreasing the fabrication of inflammatory signaling molecules and fetoprotein [133].

Phytonutrients are well-known for their beneficial effects on human health. Epidemiological and experimental studies show that dietary organosulfur compounds (OSC) play a significant role in preventing various human pathological progressions, including chronic inflammation, by decreasing inflammatory mediators such as NO, PGE2, interleukin (IL)-1, IL-6, TNF, and IL-17, which are all typical for chronic inflammation. OSC has been shown to reduce the expression of these markers, resulting in a reduction in chronic inflammatory processes [133].

#### 5.3.3. Omega-3 Fatty Acids

Some omega-3 fatty acids, such as EPA (Eicosapentaenoic acid) and DHA (Docosahexaenoic acid), are formed from the α-linoleic fatty acid, which is abundant in vegetable oil. EPA and DHA are abundantly found in fish (including fish oil) and are considered as fatty acids. They have anti-inflammatory and immune-regulatory properties by reducing leukocyte recruitment and diapedesis and limiting the synthesis of inflammatory cytokines [129].

For maintaining homeostasis and a steady internal state required for physical and mental growth, homeostasis in the levels of Omega-3 and Omega-6 concentrations is required. Adequate levels of Omega-3 are required to achieve anti-inflammatory effects, whereas Omega-6 is responsible to promote a proinflammatory state in the human body. These fatty acids act by decreasing the levels of triglycerides (TG) and cholesterol, thereby improving BMI, which results in the vasodilation of endothelial cells resulting in decreased cardiovascular risk and also improving obesity-related MeS. Additionally, secondary effects may include diminishing or decreasing insulin resistance, decreasing hypertension, and regulating lipid levels, thereby controlling dyslipidemia [130,132].

### 5.4. Dyslipidemia

Dyslipidemia is a pathological condition characterized by increased levels of lipids which includes cholesterol and triglycerides. In the human body, there are five types of lipoproteins namely VLDL (very low-density lipoprotein), IDL (intermediate density lipoprotein), HDL, Chylomicrones and LDL which are insoluble in plasma, yet they are delivered to diverse organs via lipoproteins. Lipoproteins are comprised of total cholesterol (TChol), phospholipids, triglycerides (TGs), and proteins.

#### Lipid-Lowering Plants

Various plant species including Satureja (family Lamiaceae), Teucrium (family Lamiaceae), along with traditional plants like *Glycyrrhiza glabra*, green tea, Fenugreek seeds, and *Rheum ribes* (Syrian rhubarb) are some of the plants that have been demonstrated to reduce cholesterol levels [133,134]. Various studies had confirmed the effect of *Glycyrrhiza glabra* root extract, in regulating the levels of TChol, LDL, TGs, and VLDL by diminishing the LDL oxidation susceptibility, scavenging free-radicals, and inhibiting cholesterol production [135]. Green tea has been proven to lower TChol and LDL-C levels by reducing micellar solubility and intestinal cholesterol absorption, increasing faecal fat and cholesterol excretion, lowering hepatic cholesterol levels, and up-regulating LDL-C receptors [136].

Dyslipidemia is a well-known modifiable risk factor for CVD. The use of herbal medicinal goods and supplements as an alternative treatment for a variety of metabolic diseases has exploded in popularity over the last several decades. Despite the fact that therapies utilizing a variety of plant-derived compounds have demonstrated promising success in the treatment of dyslipidemia, the question of safety remains a key roadblock. There is still a lack of understanding about the method of action, contraindications, potential adverse effects, and combinations with other medications and functional foods for the vast majority of natural products [137].

By suppressing cholesterol production, reducing oxidized LDL-C, and preventing malathion-induced activity, dried *Satureja khuzestanica* leaves have been demonstrated to be efficacious in regulating the levels of LDL, HDL, TChol, and total antioxidants. *Trigonella foenum graecum*, or fenugreek seeds, can lower TChol, VLDL, and TGs levels by multiple mechanisms, one of which is interacting with bile acids present in the gastrointestinal tract [137,138].

### 5.5. Osteoporosis

Osteoporosis is a prevalent disease among the elderly and it causes significant impairment and morbidity. Low bone density, degeneration of bone tissues, and increased fragility leading to high risk of fracture constitute the characteristic feature of osteoporosis and is often called ‘skeletal illness’. It has diverse causes depending on sex and age. Various mechanisms are associated with the development of osteoporosis including vitamin D deficiency and hyperparathyroidism. However, hormonal (estrogen) insufficiency, especially in postmenopausal women, is characterized by rapid bone turnover, leading to the development of this disorder.

Osteoporosis is characterized by a decrease in bone quality. However, there has been identification of the occurrence of Osteoporosis in patients of Type 2 Diabetes Mellitus with no drop in mineral bone density. One of the primary causes identified in such patients for their osteoporosis is the accumulation of advanced glycated end products or AGEs. It is well-known that hyperglycemia results in excessive accumulation of AGEs. In case of osteoporosis, however, it is suspected that these AGEs increase the oxidative stress and cause damage which activates the bone inflammatory response, leading to easier bone damage.

In addition, AGEs have also been reported to interfere with the proliferation of Osteoblasts and their subsequent differentiation, which is again a reported cause for MetS development. AGEs downregulate the bone metabolism by impairing the production of collagen and osteocalcin and induce apoptosis of these cells. This leads to a decrease in the production of alkaline phosphatase, which leads to an impairment of bone mineralization, and modification in extracellular matrix of these cells. This strategy has been well-established to be part of the etiology of various MetS disorders. Therefore, in conclusion, oxidative stress is enhanced by AGE accumulation, which increases osteoclastogenesis, leading to deterioration in bone quality [139].

This significant implication of oxidative stress in osteoporosis makes it another disorder falling under the umbrella term “metabolic syndrome”. However, the oxidative stress inclusion also makes management strategies for MetS disorders applicable for osteoporosis, and vice versa. Some of the treatment strategies for osteoporosis include the following:

#### 5.5.1. Lycopenes

Lycopene is basically a lipid-soluble carotenoid antioxidant with a singlet oxygen-quenching activity having 2× of β-carotene and 10× of β-tocopherol. Tomato, watermelon, and other fruits and vegetables are among its sources. As it is a molecule i.e., lipid-soluble, it requires the presence of a large quantity of lipids to be absorbed. Lycopene’s powerful antioxidant property may be responsible for the majority of its health advantages. Lycopene stimulates osteoblast cell growth and is also responsible for alkaline phosphatase activity. Lycopene activity is not only limited to osteoblast, but it also inhibits the production of ROS-secreting osteoclasts and helps in improving BMD (bone mass density) and lowers the threat of osteoporosis-associated frailty fractures and curtails N-telopeptide (NTx), a bone resorption marker [140].

The bioavailability of ingested lycopene is influenced by the amount of lycopene consumed, the interactions of lycopene with other carotenoids, and hereditary variables. Furthermore, depending on the isomeric state, the bioavailability of lycopene varies. Lycopene cis isomers are 8.5 times more bioavailable than all-trans lycopene. The enhanced solubility of cis isomers in mixed micelles accounts for their higher bioavailability compared to all-trans isomers [141].

#### 5.5.2. Phyto-Estrogens

Various active constituents present in flora, including isoflavones, lignans, flavonoids, stilbenes (resveratrol), and coumestans, constitute phytoestrogens. They exhibit pharmacological properties by binding to estrogen receptors, which act as powerful antioxidants and restrain CYP450 and aromatase enzymes. This results in inhibition of the transformation of androgens into estrogens exhibited by aromatase and has been a precursor of increased risk of breast, adrenal, and prostate cancer like disorders [142].

Plant-derived estrogenic chemicals present in natural food sources are known as phytoestrogens. Soybeans, soy products, flaxseed, and OTC (over-the-counter) dietary supplements are all good sources of phytoestrogens. Although there is evidence that phytoestrogens may have some good bone-altering effects, the full extent of their efficacy and safety has yet to be determined [142].

Isoflavonoids, flavonoids, lignans, and stilbenes are polyphenolic plant metabolites that act as phytoestrogens. Phytoestrogens have estrogenic activity and, because of their structural resemblance to 17β-Estradiol, have a greater affinity for the β-estrogen receptor. Genistein and Daidzein from soy products, Quercetin and Rutin, Resveratrol from grapes and red wine, Kaempferol and Apigenin, as well as Hesperidin, (+)-Catechin from green tea have all been shown to reduce bone loss [143].

### 5.6. Other Disorders

Hyperglycemia, hypertriglyceridemia, low HDL-C, and central adiposity are all symptoms of MeS (MeS). Because of the rise in its components, the prevalence of MeS has reached epidemic levels. The combination of Culminate MeS components could enhance the risk of T2D and CVD by five and three times, respectively. MeS is also linked to a high rate of mortality and serious cardiovascular events.

#### 5.6.1. Chromium

Trivalent chromium (Cr^3+^) is a micronutrient that is widely present in a variety of foods, including shellfish, green vegetables, and fruits. The deficiency of Cr^3+^ causes symptoms that are similar to MeS and may be associated with disorders like hyperglycemia, hypertension, and obesity (decrease in HDLs). Its mechanism of action has yet to be fully demonstrated. Cr^3+^ binds to transferrin and travels through the bloodstream. Transferrin releases Cr^3+^ and it binds to the LMWCr (low molecular weight chromium binding substance) complex in the cell, where it enhances the tyrosine kinase activity resulting in amplification of the insulin signaling pathway. Furthermore, it stimulates Glucose transporter type-4 (GLUT-4) translocation in animal muscle tissue. Although both of these processes improve insulin sensitivity, the impact of Cr^3+^ on insulin resistance in diabetics and those with MeS is contentious. These variances could be due to differences in patient’s population, dosage, and therapy length. However, multiple clinical investigations have demonstrated that it improves lipid metabolism, particularly after several months of treatment [53].

Cr^3+^ is an important mineral that appears to play a role in insulin action, MeS, and CVD regulation. There is mounting evidence that Cr^3+^ can help with insulin signaling and supplementing with it can help with systemic insulin sensitivity. There is a link between low circulating Cr^3+^ levels and the occurrence of T2D, as diabetes patients have lower tissue Cr^3+^ levels than healthy controls [144].

#### 5.6.2. Cinnamon

Although the specific MoA of aqueous cinnamon extract is unknown, it has been shown to promote autophosphorylation, which leads to both insulin receptor activation and tyrosine phosphatase inhibition. Its antihypertensive, anti-triglyceride, and anti-diabetic effects have also been documented in the preclinical and clinical studies as well [145,146,147]. In animal tests, it increased glucose absorption, is involved in the action of glycogen synthase enzyme, and is also effective against insulin resistance. The ligand-like activity mediated by cinnamon is similar to thiazolidinedione (TZDs), along with the activator receptors for peroxisome proliferator activated receptor (PPAR), specifically PPAR-α and PPAR-β. This led to the conclusion that cinnamon exerts activity like PPAR agonists and can enhance the maturation of adipocytes [148].

Cinnamon and its active components, such as cinnamaldehyde, cinnamic acid, and eugenol etc., in the form of aqueous and alcoholic extracts have a variety of medicinal effects. Cinnamon extracts help with a variety of MeS symptoms, including excessive blood glucose, dyslipidemia, obesity, and high blood pressure. It has been found to be a cardiovascular protective agent with the potential to reduce MeS consequences due to its anti-diabetic, antioxidant, anti-inflammatory, and lipid profile-beneficial properties [149].

Cinnamon and its constituents have been proven to have favorable benefits on almost every aspect of MeS, including insulin sensitivity, glucose regulation, production of antioxidants, role in inflammation, blood pressure regulation, and bodyweight management. In vitro studies have also indicated that cinnamon and its components enhance factors linked to diseases like Alzheimer’s, stroke, and cancer [149].

**Figure 7:**
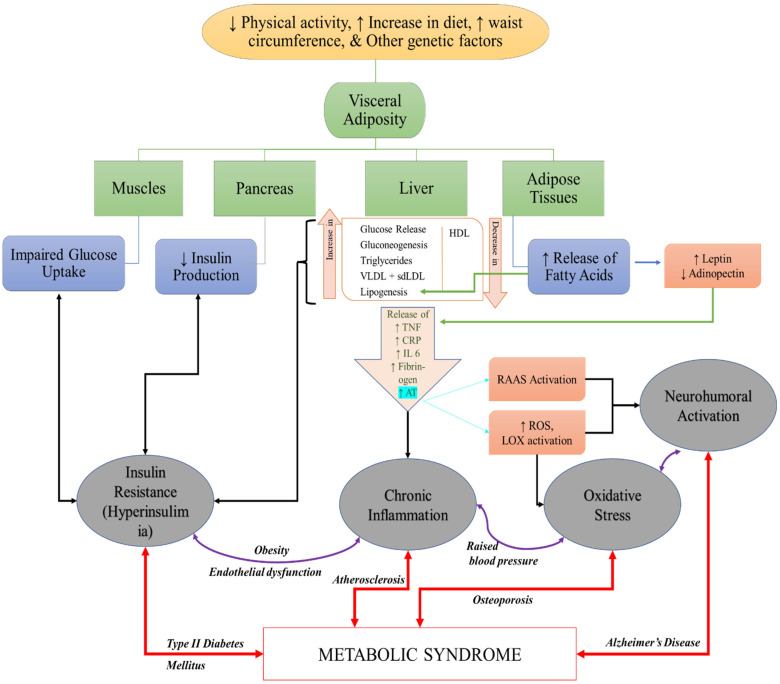
A summary of the reported pathophysiology of Metabolic syndrome. With a variety of activating factors, most of the symptoms end up contributing to either a Insulin Resistance, b Chronic Inflammation, c Oxidative Stress or d Neurohumoral activation. These four strategies are also inter-linked, demonstrated by the presence of two-sided arrows between them. Therefore, RCS becomes a universal factor in activating more than one causative agent that contribute to Metabolic Syndrome, making it one of the key attributes to be acknowledged in developing management strategies against RCS. (VLDL = Very Low Density Lipoprotein, HDL = high Density Lipoprotein, sdLDL = Small dense Low Density Lipoprotein, TNF = Tumor Necrosis Factor, CRP = C Reactive Protein, IL 6 = Interleukin 6, AT = Angiotensin, RAAS = Renin Angiotensin Aldosterone System, ROS = Reactive Oxygen Species, LOX = Lipoxygenase)**.**

The detailed representation (Figure 7) of the mechanistic approach of various factors which includes lifestyle practices such as physical activity, diet, waist circumference along with genetic factors play a key role on the trigger involved in the pathogenesis of MeS. The major four’s which includes muscle pancreas liver and adipose tissue are directly and indirectly involved in insulin resistance, chronic inflammation, oxidative stress and neurohumoral activation. The role of muscle and the pancreas in impaired glucose uptake and decreasing insulin production leads to insulin resistance, resulting in the development of hyperinsulinemia that ultimately leads to MeS. Moreover, liver and adipose tissue are responsible for maintaining homeostasis in glucose maintenance and indirectly involved in RAAS activation along with increased ROS and LOX (Lipoxygenase) activation pathways. These down regulatory pathways are involved in increasing oxidative stress and through RAAS activation may lead to chronic inflammation as well. The combination of chronic inflammation and oxidative stress is associated with atherosclerosis and osteoporosis. Increased oxidative stress due to ROS and LOX activation leads to the development of neurohumoral activation, which further leads to the development of Alzheimer’s disease. The combinations of insulin resistance and chronic inflammation or oxidative stress and neuro humoral activation are associated with the pathogenesis of various disorders mentioned in Figure 7 and ultimately contribute to the pathogenesis of MeS.

## 6. Discussion and Conclusions

From the review of literature and analysis carried out in this review article, it can be postulated that MeS is a disorder of concern and may even be considered a lifestyle disease [150]. More than 25% of the population in the industrialized nations are reported to have this MeS leading to increased mortality and morbidity. With lifestyle changes, the percentage of people affected with this syndrome might increase, and it may be considered that it could become an epidemic of general public health concern. Various studies have concluded that MeS is associated with an array of pathological conditions, including those of cardiovascular origin, neurological disorders, and even metabolic disorders, including diabetes, obesity, dyslipidemia, and osteoporosis as well [151].

The pathologies of these disorders are characterized by increased inflammation, oxidative stress, and hypoxia, which are considered the critical mediators in developing various complications [152]. In this article, we have also highlighted the important role played by RCS species in propagating MeS disorders, followed by strategies that can be used to combat them. From the research carried out in the past few decades, it can be confirmed that various synthetic and natural agents are available which have a strong affinity to target carbonyl species in the management of metabolic disorders.

These agents have provided promising results in preclinical studies that include in vitro and in vivo data which can provide all benchmarks to further clinical studies. These compounds are associated with the regulation of various cells including immune cells (T-cells, B-cells, killer cells, etc.) and associated with various pathways, including NO production, NF-κB (nuclear factor kappa light chain enhancer) activation, and iNOS pathway. In this review article, we have highlighted a few natural and synthetic agents that have shown activity in regulating these pathways and can become a drug of choice in combination with other therapeutic agents in the effective management of various pathological conditions. The therapies that show most promise include synthetic agents carnosine and simvastatin and natural agents such as polyphenols. However, there is still a long way to go before their efficacy is established and there are data specific to the disorder they will be used to treat. However, their roles in RCS scavenging make them very viable candidates.

It is suggested from the available literature data that the therapeutic strategy targeting this RCS may provide multidisciplinary interventions in the management of various pathological conditions. This is because the underlying disease etiologies for most of the MeS disorders are very similar, allowing for the use of multiple agents for similar disorders.

Due to this pandemic, people have started improvising lifestyle and dietary changes and have realized the importance of these factors in the management of various complications including MeS. These therapeutic agents along with the combination of the above-mentioned factors can help in combating various metabolic disorders.

## Figures and Tables

**Table 1:** Various studies carried out for exploring the effect of RCS in preventing MeS. RAGE (Receptor of AGE protein adducts), AGEs (Advanced Glycation Endproducts), MG (Methylglyoxal) and HNE (4-Hydroxy-Trans-2-NonEnal). These are the RCS targeted using the mentioned targeted agents and experimental models. These studies implicate the role RCS in pathogenesis of various MeS.

Metabolic Disorders	RCS	Targeted Agent	Experimental Model	References
In-vitro Study				
Diabetic nephropathy	RAGE	Glucagon-like peptide	Human mesangial cells	[49]
Insulin resistance	AGEs, Protein carbonyls	NAC, AGD, SAM, D3T	Striated Gastrocnemius muscle	[50]
Human Study				
Diabetes	MG	Metformin	Type II diabetes patients	[51]
Diabetes complications	RAGE	Simvastatin	Type II diabetes patients	[52]
In-vivo Study				
Liver damage	AGEs, RAGE, protein carbonyls	Glycyrrhiza	Wistar rats fed with high fructose	[53]
Dyslipidemia	HNE (4-Hydroxy-trans-2-NonEnal), AGEs	Carnosine	Zucker rats	[54]
Diabetic atherosclerosis	RCS, AGEs, ALEs, RAGE	LR-90	Diabetic rats induced with streptozotocin	[55]
Diabetic neuropathy	RAGE	Candesartan	Dahl salt-sensitive rats injected with MG	[56]
Liver/renal toxicity	RAGE, protein carbonyls	Prach	Wistar rats injected with CCl_4_	[57]
Type II Diabetes	RCS, HNE, PUFAs	Carnosine	Fructose fed Wistar rats and HFHS fed GP 4 het mice	[47]

**Table 2:** Various studies targeting RCS for management of metabolic diseases.

Metabolic Diseases	RCS	Targeted Agent	In-Vivo Model	Reference
Dyslipidemia	ALEs, AGEs, RCS, HNE	Carnosine	Zucker obese rats	[47]
Renal function	ALEs, AGEs, RCS, HNE	Carnosine	Zucker obese rats	[47]
Obesity	HNE, RCS, AGE, PUFAs	Carnosine	Fructose-fed rats	[72]
Anticancer	AGEs, ALEs, RCS	MGO	Mitochondria malignant cells	[72]

**Table 3:** Use of carnosine in various MeS and its role on various MeS and results from various studies that target obesity, dyslipidemia, and renal function using the mentioned models.

MeS	Drug	Model	**Result**	Reference
Obesity	Carnosine	Obese Humans	Significant decline in the percentage of egested adducts followed by a significant elevation of the urinary excretion of carnosine (through urine)	[74]
Dyslipidemia	Carnosine	Zucker obese rats	In obese Zucker rats, both L- and D-CAR (D-carnosine) dramatically reduced obesity-related illnesses by preventing the development of dyslipidemia, hypertension, and kidney damage.	[47]
Renal Function	Carnosine	Zucker obese rats	In obese Zucker rats, both L- and D-CAR dramatically reduced obesity-related illnesses by preventing the development of dyslipidemia, hypertension, and kidney damage.	[47]

## Data Availability

Not applicable.

## Abbreviations

AGE	Advanced glycation end products
ALE	Advanced Lipoxidation end products
APO	Apolipoprotein
BMI	Body Mass Index
CAT	Chloramphenicol acetyltransferase
CNDP 1	Carnosine Dipeptidase 1
CYP450	Cytochrome P450
Cr+3	Trivalent chromium
CVD	Cardiovascular disease
DHA	Docosahexaenoic acid
DNA	Deoxyribonucleic acid
EPA	Eicosapentaenoic acid
GABA	Gamma-Aminobutyric acid
GLUT 4	Glucose transporter type-4
GSH	Peroxide Glutathione peroxide
HDL-C	High density lipoprotein- Cholesterol
HMG CoA	β-Hydroxy β-methyl glutaryl-CoA
HNE	4-Hydroxy-trans-2-NonEnal
LDL-C	Low density lipoprotein-Cholesterol
LMWCr+3	Low molecular weight chromium binding
LOOH	Lipid peroxides
LP	Lipoprotein
LP-a	Lipoprotein a
MetS	Metabolic syndrome
mRNA	Messenger RNA
NrF2	Nuclear factor erythroid 2-related factor 2
NTx	N-elopeptide
PPAR	Peroxisome proliferator-activated receptor
RCS	Reactive carbonyl species
ROS	Reactive oxygen species
Tchol	Total cholesterol
TGs	Triglycerides
TNF	Tumor necrosis factor
TZDs	Thiazolidinedione
T2D	Type-2 diabetes
